# Food availability before aestivation governs growth and winter reproductive potential in the capital breeding fish, *Ammodytes japonicus*

**DOI:** 10.1371/journal.pone.0213611

**Published:** 2019-03-07

**Authors:** Hirotsune Kuzuhara, Michio Yoneda, Tatsuo Tsuzaki, Masanori Takahashi, Naoaki Kono, Takeshi Tomiyama

**Affiliations:** 1 Graduate School of Biosphere Science, Hiroshima University, Higashi-Hiroshima, Hiroshima, Japan; 2 National Research Institute of Fisheries and Environment of Inland Sea, Japan Fisheries Research and Education Agency, Imabari, Ehime, Japan; 3 National Research Institute of Fisheries and Environment of Inland Sea, Japan Fisheries Research and Education Agency, Hatsukaichi, Hiroshima, Japan; Swedish University of Agricultural Science, SWEDEN

## Abstract

Capital breeders develop gametes by using energy that was stored before the spawning season. Energy is allocated to growth and reproduction, and limited food availability affects the balance of energy allocation, especially in fish that mature within a year, such as western sand lance (*Ammodytes japonicus*). This species aestivates without feeding until winter spawning and utilize energy stores that were accumulated prior to aestivation for maturation and spawning. This study aimed to evaluate the growth, energy storage, maturation rate, and reproduction of *A*. *japonicus* in response to food availability before aestivation. We conducted laboratory experiments in which young-of-the-year *A*. *japonicus* were fed at rates of 4% and 1% of their body weight per day; assigned as high and low ration groups, respectively. In June, body length was found to be significantly larger in the high ration group than in the low ration group, but the somatic condition did not differ significantly between the groups. Maturation rates and average fecundities were 1.0 and 6297 in the high ration group and 0.8 and 2251 in the low ration group, respectively. These results indicate that food availability before aestivation strongly governs the reproductive potential of *A*. *japonicus*, and suggest the involvement of mechanisms in the inter-annual recruitment variation in sand lance species.

## Introduction

Energy allocation for metabolism, somatic growth, and maturation is a key issue related to optimal reproduction in fish [1,2]. During early life stages, surplus energy is allocated primarily to somatic growth, and this growth-biased allocation results in the reduction in size-related mortality [3–5]. Somatic growth also affects the timing of maturity [6] and fecundity [7]. Sufficient growth and energy accumulation are essential for successful reproduction.

Fishes adopt various reproductive strategies, such as income-capital strategies, in order to more efficiently increase the number of offspring produced in their life-time [8,9]. Income and capital breeders are different in their timing of energy acquisition for use in egg production. Income breeders use energy acquired during the spawning season, and their feeding during this season affects their reproductive investment [10]. Their oocytes develop asynchronously, and females spawn multiple batches during a relatively long spawning season. On the other hand, capital breeders use energy that is stored before the spawning season. Their oocytes develop synchronously, and females spawn once or several times during a spawning season. Fecundity is regulated according to the nutritional status of the parent fish, as reported for Atlantic herring *Clupea harengus* [11], Atlantic cod *Gadus morhua* [9,12], and European plaice *Pleuronectes platessa* [13]. Therefore, food availability before the spawning season is expected to affect the reproductive traits of capital breeders.

Sand lances (or sand eels, genus *Ammodytes*) are capital breeders and commercially and ecologically important species because of the key roles they play as forage fish. They serve as a high-lipid food source [14], and their abundance often affects the breeding of predatory seabirds [15,16] or the nutritional status of piscivorous fishes [17]. Body size, growth, age at maturity, and fecundity of sand lances have been shown to vary between regions or years [18–22]. The relationships between food availability and reproductive potential or other biological traits of the sand lance are important for effective and sustainable stock management.

Phenology of energy accumulation, maturation, and gonadal development are highly variable between sand lance species. Some *Ammodytes* species exhibit active feeding from May to September and subsequently spawn from September (*Ammodytes dubius* [23]; *Ammodytes hexapterus* [24,25]), with their gonads developing during feeding periods [23]. Other species, such as lesser sandeel *Ammodytes marinus* and western sand lance *Ammodytes japonicus* (formerly known as *A*. *personatus*, revised by [26]) have developed a unique habit of resting in the sand to reduce their metabolism before spawning [27,28]. Specifically, *A*. *marinus* exhibits high feeding activity from April to June [29], and rests in the sand from September to March, except during spawning in December and January [22,30,31]. Similarly, *A*. *japonicus* grows up until June or July and thereafter aestivates until spawning from December to January [32,33]. The timing of the onset of gonadal development differs between *A*. *marinus* (from June to August [34]) and *A*. *japonicus* (from November [32,35]). However, both species accumulate energy (for reproduction in winter) during a short foraging period before aestivation. Thus, food shortage prior to aestivation would affect the reproduction of these species.

Western sand lance is distributed along the coast of Japan. In the Seto Inland Sea, western Japan, this species usually matures at 1 year of age, and although their life span is ~3 years, a large portion of parental fish are 1 year old (55% of spawning stock biomass in 1956–1964 and 62% in 2011–2016) [36,37]. The stock of this species declined from 123 thousand tons in 1992 and reached historically low level (2 thousand tons) in 2017 due to the lowest abundance of recruit fish (Takahashi M. and Kono N., unpublished data). While there seems to be no significant relationship between the abundance of recruit fish and spawning stock biomass over the last several decades [37], understanding the reproductive potential of first spawning (1-year-old) fish could be the key to elucidate factors causing the recent reduction in the stock.

The main purpose of this study is to explore the effects of food availability before aestivation on maturation and egg production of first-spawning western sand lance under captive condition. Because this species does not feed during aestivation, the energy for reproductive investment should be determined at the onset of aestivation, around June. This species matures only if the condition factor (Fulton’s K) exceeds 4.2 before aestivation [38]. To achieve this condition, sand lance need to allocate more energy to somatic growth (body weight) than skeletal growth (body length). However, body length directly governs the fecundity of this species [32,38]. Therefore, sufficient somatic growth and skeletal growth prior to aestivation are ideal for young-of-the-year (YOY) fish, but if food availability is limited, individuals need to adjust their energy allocation to either improve the somatic condition or increase the body length. From these, YOY fish were reared at ample and limited food conditions before aestivation to monitor growth trajectories, and thereafter they were kept in tanks with sand beds under aestivation to compare reproductive traits between the two treatments. We addressed the following questions: (1) How do somatic and skeletal growths of YOY fish respond to the different food availabilities? (2) Does food availability before aestivation affect reproductive potential of females? (3) How do females previously experienced a nutritionally harsh environment adjust their energy to invest the egg production?

## Materials and methods

### Preparation of laboratory experiments

YOY western sand lance were caught by a commercial two-boat seine in Itsukinada, western Seto Inland Sea (33° 57' N, 132° 44' E) in April 2016 and were transported to the Hakatajima Station, National Research Institute of Fisheries and Environment of Inland Sea. Study animals (~300 individuals) were maintained in each of the four 1500 L tanks filled with running seawater. No sand was added to the tanks. Pellets (Alteck, 1.0–1.5 mm in particle diameter, Marubeni Nisshin Feed Co., Ltd.) were fed to the fish ad libitum during the daytime for 3 weeks to allow for acclimation to the laboratory conditions. The water temperature ranged from 13.1°C to 14.7°C during the acclimation period.

### Experiment I: Effect of food availability on growth before aestivation

YOY western sand lance [n = 200, 70.7 ± 6.7 mm (mean ± SD) standard length (SL)] were housed per 1000 L circular tank. Six tanks were used, three of which were assigned to the high ration group and the remaining three were assigned to the low ration group. Each tank was filled with running seawater and contained a sand bed (sand: approximately 1 mm in diameter; depth: ~5 cm) that was constructed using a plastic container (50 × 32 × 9 cm, length × width × height). Fish were subject to natural seawater without temperature adjustment, and water temperature was recorded every 30 min in each tank using a temperature logger (Tidbit V2, Onset). According to the relevant food-manipulated experiments [38], fish were fed with pellets at rates of 4% (under satiation) and 1% of their average body weight (BW, g) per day in the high and low ration groups (hereafter referred to as HR and LR), respectively. Fish were fed four times per day using an automatic feeder (DF-100MS, Chubu Kaiyo Kaihatsu Co., Ltd.). The amount of food provided was appropriately adjusted based on the BWs measured every three weeks.

The experiment was conducted for nine weeks from April 28 to June 30, 2016. Specimens were sampled and measured every three weeks from the day prior to the start of the experiment. When extracting specimens, all individuals that buried themselves in the sand were collected from the sand bed and 8 to 10 specimens were randomly selected. Specimens were anesthetized with 300 ppm 2-phenoxyethanol, sacrificed, and then measured (i.e., SLs and BWs).

### Experiment II: Effect of food availability on maturation and reproduction

Similar to Experiment I, we used four 1000 L tanks that were assigned to the HR (two tanks) and LR (two tanks) groups, and study animals were fed 4% and 1% of their BW per day, respectively. Approximately 300 individuals were accommodated per tank on May 2, 2016. Fish were subject to natural seawater (ambient) temperature throughout the experiment. Fish were fed four times per day using an automatic feeder. The feeding amount was appropriately adjusted according to individual growth and survival rates. Water temperature was recorded every 30 min using a temperature logger (Tidbit V2). To examine the body size and somatic condition before aestivation, 30 fish per ration group were randomly collected and their SLs and BWs were measured on July 14. Thereafter, a sand bed was placed in each tank, and feeding was discontinued. Most individuals buried themselves in the sand bed on July 16 and were considered to have entered aestivation. The water temperature was 22.4°C on July 16. Thereafter, two tanks for each ration group were pooled by transferring one sand bed to the other tank, because no significant effect of ‘tank’ was observed for SL (two-way analysis of variance [ANOVA], *F*_2,56_ = 1.53, *p* = 0.23) or BW (*F*_2,56_ = 0.98, *p* = 0.38) on July 14.

Approximately 10 to 19 individuals were extracted from each ration group and sacrificed for measurements of SL and BW on November 16 and 30, December 12 and 26, and January 11. The maturity or immaturity and sex (only for mature individuals) were identified by visual inspection of the gonads. For mature females, wet weight of the ovaries was measured to the nearest 0.01 g. Ovaries were fixed with 10% formalin, and subsamples (0.1–0.2 g) were collected from the center of the right ovary of each individual to estimate the egg density (N g^−1^) and fecundity. The effect of formalin preservation on the gonad weight was considered negligible. The number of oocytes in each subsample was counted, and oocyte diameter was measured for 30~50 oocytes per individual fish under a microscope. These procedures were conducted for mature females sampled on December 12 and 26, and January 11. Individuals collected in November were not sufficiently mature for oocyte diameters to be measured, especially in the LR group.

### Analyses

For both Experiments I and II, the somatic condition of western sand lance was evaluated by Fulton’s condition factor K, following previous studies on this species [33,38]: K = BW × 10^6^ × SL^−3^.

In Experiment I, one-way nested ANOVA was used to test the differences between SL, BW, or K between the ration groups on each sampling date, and the tank was considered to be a random factor. To explore temporal changes, Tukey-Kramer multiple comparison test was operated for SL, BW, or K between the ration groups and dates. In this test, the effect of tank was not considered.

In Experiment II, the reduction in BW through aestivation was compared between the ration groups using the following method: a linear model for log (BW) was constructed, wherein the log (SL), month (July and November), ration group (HR and LR), and the interaction between month and ration group were used as the initial explanatory variables. The reduction rate from July to November was assessed from the coefficient of the ‘month’ (November). As the reduction rate differed between the ration groups, the interaction of ‘month’ and the ration group was also included in the model. The model was selected through stepwise selection based on the Akaike information criterion (AIC).

To explore the factors that may explain the maturity (or immaturity) of western sand lance, a generalized linear model (GLM) with binomial error and logit-link function was used. Because the maturation rate changed until December 12 in the HR group (see results), the maturity of individual fish (mature or immature) sampled on December 12, 26, and January 11 was used as the response variable. Because all individuals from the HR group matured from December 12 (see results), the GLM was constructed only for the LR group. Initial explanatory variables used were sampling date and the SL. BW or K were not included because of collinearities with SL. The model was selected through stepwise selection based on the AIC.

Oocyte diameter was compared between ration groups. One-way nested ANOVA was used for each date, and female individuals were considered as a random factor. Preliminarily, a linear mixed model was conducted for oocyte diameter. Initial explanatory variables were the sampling date, ration group, and SL of females, and female individuals were used as a random factor. The SL was excluded and others were included as explanatory variables in the selected model based on the AIC.

Fecundity of mature females was estimated using the gravimetric method based on the egg density (N g^−1^) of the subsample: fecundity = ovary weight × egg density. Relative fecundity (fecundity × BW^−1^) was calculated for each ration group. Individuals collected on December 26 and January 11 were used, because gonads were not sufficiently developed until December 12 (see results), which may have affected the relative fecundity due to greater BW. Preliminarily, we confirmed that the egg density did not vary between the right or left ovaries nor between the position of the ovaries by assessing three random females from each of the HR and LR groups on December 26 and January 11. Approximately 0.1–0.2 g of ovary subsamples were extracted from each part (one third from the anterior, one third from the posterior and one third from the center) of the left and right ovaries. We constructed a linear mixed model in which egg density was used as a response variable. Ration groups, left or right ovaries, and position (anterior, middle, or posterior) were used as the initial explanatory variables. Individual was used as the random variable. The final model was determined according to the Bayesian information criterion. All explanatory variables were excluded from the model, which indicated that variations in egg density were negligible.

To test whether fecundity and relative fecundity differed between the ration groups, linear models were constructed. Because fecundity is an allometric trait that is generally described by a power function of SL [22, 38], log (fecundity) or log (relative fecundity) were used as the response variables and log (SL), ration group, and the interaction thereof were used as the initial explanatory variables. Models were selected through stepwise selection based on the AIC.

All experimental procedures followed the guidelines for animal welfare of Fisheries Research and Education Agency, Japan (50322001) and were approved by the committee of animal welfare of the National Research Institute of Fisheries and Environment of Inland Sea (no. 2016–8).

## Results

### Experiment I

The study animals readily consumed their feed and showed negligible mortality in both treatment groups. Most of the individuals in the HR group entered aestivation before the end of June while most of the individuals in the LR group were still seen swimming in the water column in all three LR tanks on June 30.

**Fig 1:**
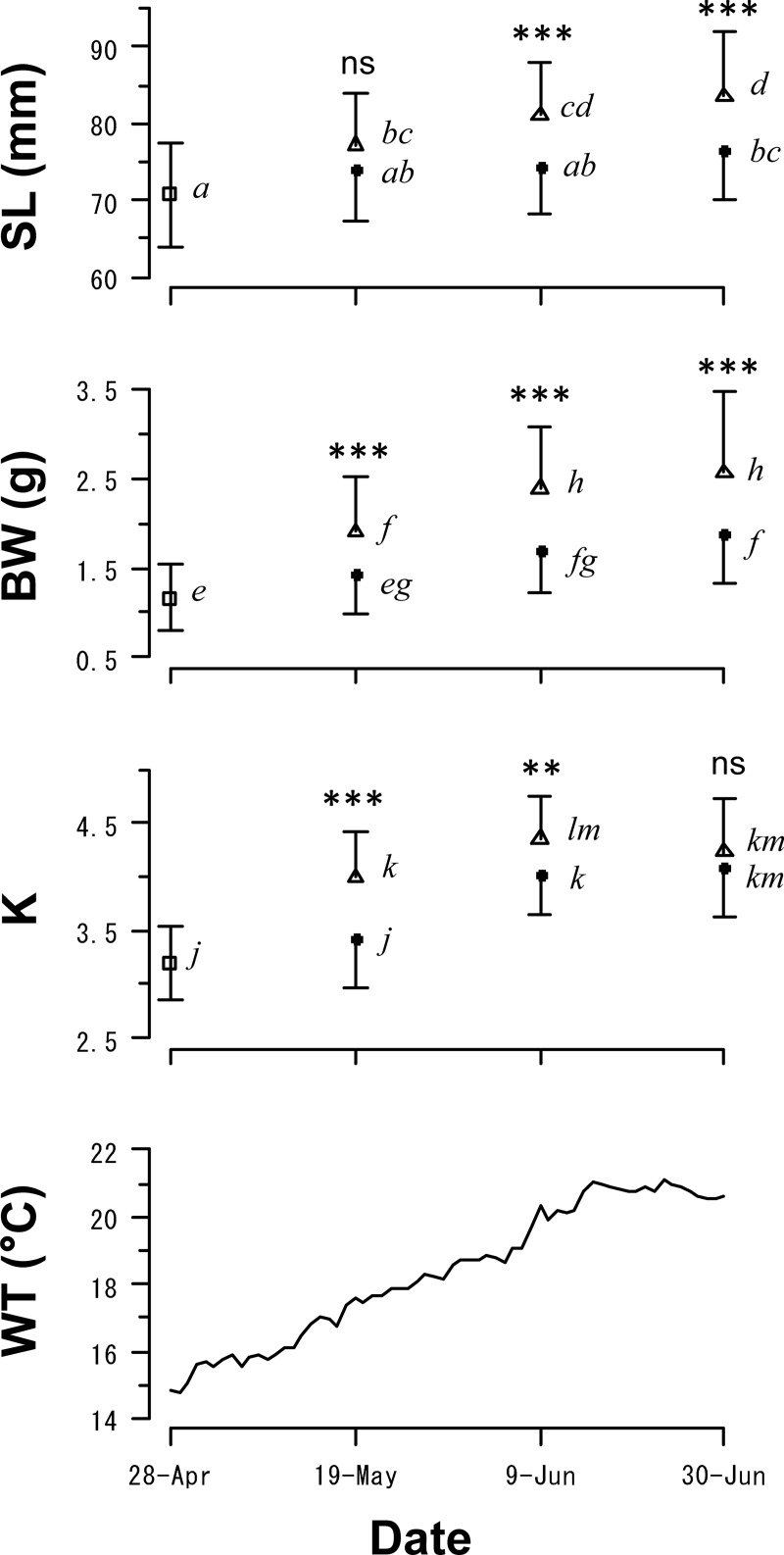
Temporal changes in the standard length (SL), body weight (BW), and condition factor (K) of western sand lance (*Ammodytes japonicus*) and associated water temperature (WT) in Experiment I. Open triangles and solid circles indicate high and low ration groups, respectively. Open squares indicate values at the beginning of the experiment. Data represent mean and standard deviation. Differences in SL, BW, and K between the ration groups were tested using one-way nested ANOVA (*: *p* < 0.05, **: *p* < 0.01, ***: *p* < 0.001, ns: not significantly different). Different italicized letters indicate significant differences between groups (Tukey-Kramer test, *p* < 0.05).

The SLs of individuals in the HR group increased throughout the experiment while those of the individuals in the LR group remained almost unchanged from May 19 to June 30 despite the relatively high water temperature (≥17.5°C, Fig 1). There were significant differences in the SLs between the two treatments after June 9 (nested ANOVA, June 9: *F*_1,51_ = 14.28, *p* < 0.001; June 30: *F*_1,127_ = 30.37, *p* < 0.001). On the other hand, the BWs of individuals in both treatments significantly increased from April 28 to June 9, and significant differences were consistently observed between treatments (May 19: *F*_1,54_ = 13.37, *p* < 0.001; June 9: *F*_1,51_ = 19.94, *p* < 0.001; June 30: *F*_1,127_ = 33.33, *p* < 0.001). The final SLs at the end of the experiment on June 30 were: 83.6 ± 8.2 mm (n = 52, mean ± SD) for the HR group and 76.5 ± 6.6 mm (n = 81) for the LR group. The final BWs at the end of the experiment were: 2.56 ± 0.89 g for the HR group and 1.88 ± 0.55 g for the LR group.

The K of HR increased until June 9 (4.36 ± 0.39) and then slightly decreased. This decrease in K was due to the onset of aestivation in some specimens from June 9. No individuals in the HR group were observed swimming in the water column in two of the three tanks on June 30. The K was observed to rapidly increase from May 19 to June 9 in specimens in the LR group (Tukey-Kramer test, *p* < 0.001). Significant variations in K were observed between treatments from May 19 to June 9 (May 19: *F*_1,54_ = 26.48, *p* < 0.001; June 9: *F*_1,51_ = 11.07, *p* < 0.01), however, no significant differences in K were observed at the end of the experiment (*F*_1,127_ = 3.36, *p* = 0.069).

### Experiment II

The SLs differed significantly between the HR and LR groups (two-way ANOVA, *F*_1,93_ = 181.28, *p* < 0.001) but were similar between before and after aestivation (July vs. November, *F*_1,93_ = 0.17, *p* = 0.68, Table 1), and no significant interaction was observed between the ration groups and sampling months (*F*_1,93_ = 0.31, *p* = 0.58). The somatic condition (i.e., the condition factor K) had decreased greatly after aestivation in both ration groups. Both log (SL) and month were adopted as explanatory variables in the linear models for log (BW) in the HR and LR groups (Table 2). The reduction rate during aestivation was estimated as 0.356 and 0.422 in the HR and LR groups, respectively.

**Table 1:** Standard length (SL), body weight (BW), and Fulton’s condition factor (K) of western sand lance (*Ammodytes japonicus*) in July (July 14) and November (November 16 and 30) in Experiment II.

Month	Group	N	SL (mm)	BW (g)	K
July	High ration	30	97.98 ± 8.46	5.02 ± 1.37	5.22 ± 0.30
	Low ration	30	76.90 ± 6.43	2.03 ± 0.60	4.35 ± 0.37
November	High ration	18	98.53 ± 10.60	3.70 ± 1.30	3.69 ± 0.52
	Low ration	19	75.59 ± 6.32	1.27 ± 0.44	2.86 ± 0.44

Values indicate means ± SDs.

**Table 2:** Results of the linear model for log (body weight) of western sand lance (*Ammodytes japonicus*) in Experiment II.

Analysis of variance				Summary			
Error source	SS	df	*P*	Variable	Estimate	SE	*p*
Error	1.10	45		Intercept	-13.66	0.58	<0.001
Log (SL)	8.23	1	<0.001	Log (SL)	3.33	0.13	<0.001
Month	3.47	1	<0.001	Month (Nov)	-0.356	0.033	<0.001
Group	0.13	1	0.0014	Group (LR)	-0.11	0.042	0.014
Month: Group	0.025	1	0.15	Month (Nov): Group (LR)	-0.066	0.046	0.15

Analysis of variance was operated by Type II F test.

Adjusted R-squared = 0.97.

SS: sum of squares.

Initial explanatory variables were log (standard length [SL]), month (July and November), ration group (high ration [HR] and low ration [LR]), and the interaction between month and ration group.

Month (Nov): the effect of November that was assessed on the basis of July.

Group (LR): the effect of low ration (LR) that was assessed on the basis of high ration (HR).

All explanatory variables were selected based on Akaike information criterion.

**Fig 2:**
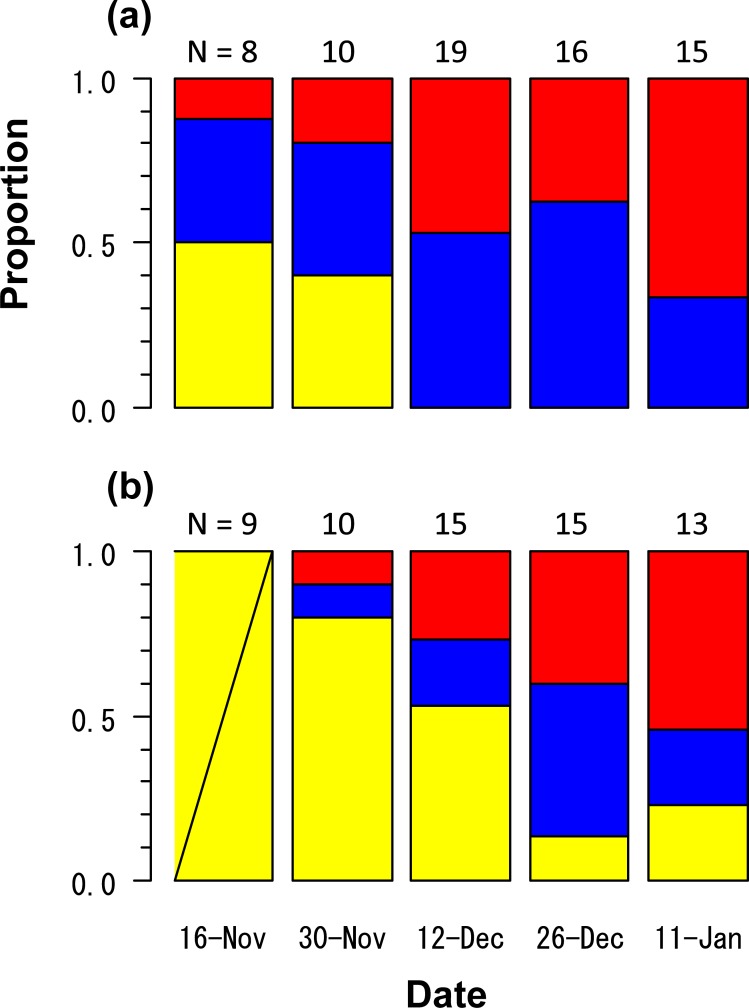
**Temporal changes in the proportion of mature and immature western sand lance (*Ammodytes japonicus*) in the (a) high ration and (b) low ration treatments in Experiment II.** Yellow, blue, and red bars indicate immature individuals, mature males, and mature females, respectively. Numerals indicate sample sizes.

**Fig 3:**
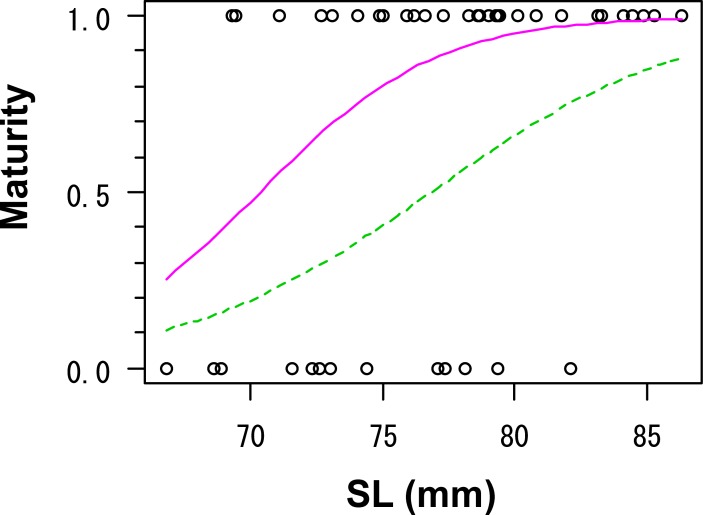
Relationship between standard length (SL) and the maturity of western sand lance (*Ammodytes japonicus*) in the low ration treatment in Experiment II. Specimens collected on December 12, 26, and January 11 were used. Binomial maturity (mature = 1, immature = 0) assigned to each individual was plotted. The green dashed line represents the generalized linear model (GLM; logit [Maturity] = 0.21 × SL– 16.12) fitted to the data of December 12. The purple solid line represents the GLM (logit [Maturity] = 0.30 × SL– 21.21) fitted to the pooled data of December 26 and January 11.

The maturation rates of western sand lance increased from November and reached 1.0 in the HR group on December 12 (Fig 2). The sex ratio was not biased because of an equivalent number of individuals (25 males and 25 females) from December 12 to January 11. In the LR group, the maturation rate also increased from 0 on November 16 to 0.87 on December 26. All explanatory variables (SL, date, and treatment) were adopted in the GLM for maturity of the LR group based on the AIC, when using the dataset of three dates from December 12 to January 11 (Table 3). However, the 'date' was excluded from the GLM using the dataset of two dates of December 26 and January 11 (Table 3). The SL at 50% maturation was estimated to be 76.8 mm for December 12 and 70.4 mm for December 26 and January 11 (Fig 3). On the other hand, all individuals from HR group (females: 71.9–115.5 mm SL; males: 77.4–114.1 mm SL) matured during the period from December 12 to January 11.

**Table 3:** Results of the generalized linear model for maturity (mature/immature) of western sand lance (*Ammodytes japonicus*) in low ration group in Experiment II.

Analysis of deviance				Summary			
Error source	SS	df	*P*	Variable	Estimate	SE	*p*
For December 12, 26, and January 11							
Error	41.14	39		Intercept	-16.12	7.11	0.023
SL	6.42	1	0.018	SL	0.21	0.093	0.024
Date	5.35	2	0.092	Date (Dec 26)	2.03	1.01	0.044
				Date (Jan 11)	1.43	0.93	0.12
For December 26 and January 11							
Error	25.75	26		Intercept	-21.21	10.93	0.052
SL	6.37	1	0.018	SL	0.30	0.15	0.041

Analysis of deviance was operated by Type II F test.

SS: sum of squares.

Initial explanatory variables were standard length (SL) and date.

The effect of date was assessed on the basis of December 12.

All explanatory variables were selected based on Akaike information criterion.

**Fig 4:**
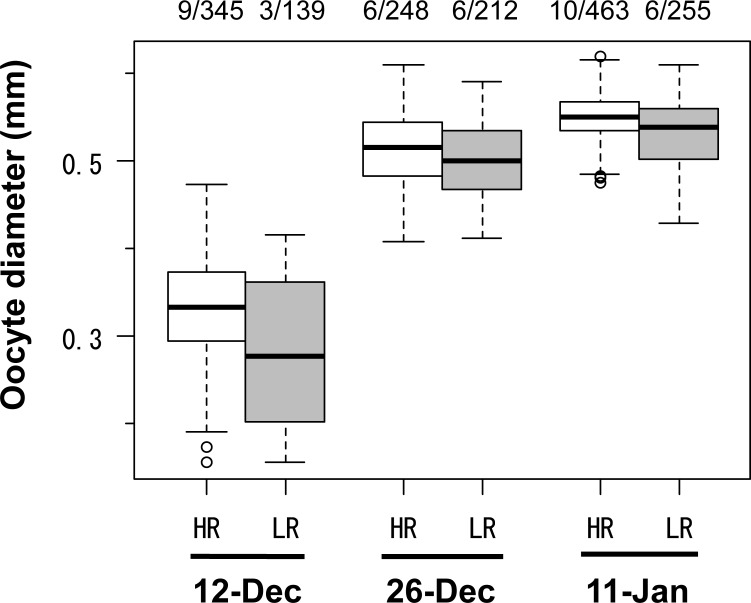
Boxplots of the oocyte diameter in western sand lance (*Ammodytes japonicus*) in Experiment II. Numerals indicate the number of individuals / the number of eggs measured. Significant differences between the high ration (HR) and low ration (LR) groups were detected (one-way nested ANOVA, *p* < 0.001 for all dates).

Gonad development was also delayed in individuals in the LR group compared to those in the HR group. Male spermiation was observed in the HR and LR groups on November 30 and December 12, respectively. Oocyte diameters were 0.33 ± 0.04 mm (n = 9, mean ± SD) and 0.25 ± 0.09 mm (n = 3) in the HR and LR groups, respectively, on December 12 (Fig 4). Oocyte diameters thereafter increased to 0.51 ± 0.03 mm (n = 6) and 0.50 ± 0.03 mm (n = 6) on December 26, and 0.55 ± 0.01 mm (n = 10) and 0.53 ± 0.03 mm (n = 6) on January 11 in the HR and LR groups, respectively. Oocyte diameters were significantly larger in the HR group than in the LR group (one-way nested ANOVA, December 12: *F*_1,472_ = 172.0, *p* < 0.001; December 26: *F*_1,448_ = 26.83, *p* < 0.001; January 11: *F*_1,702_ = 105.15, *p* < 0.001).

**Fig 5:**
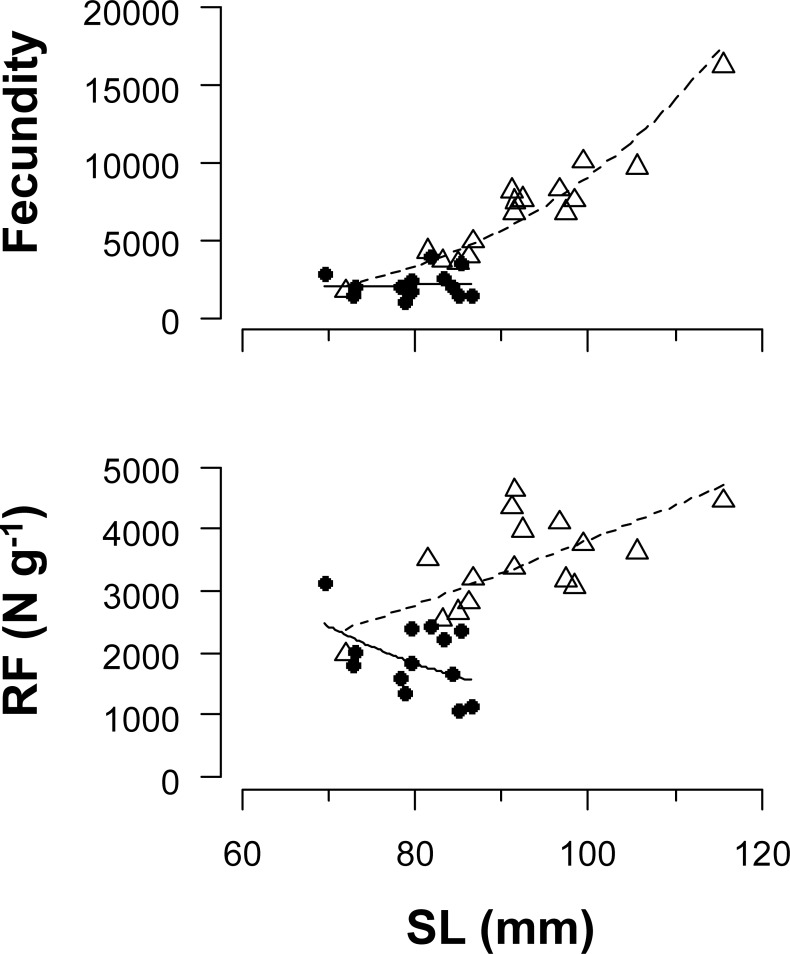
Relationship between standard length (SL) and fecundity or relative fecundity (RF: Number of oocytes per unit body weight) in western sand lance (*Ammodytes japonicus*) in Experiment II. Individuals collected on December 26 and January 11 were used. Open triangles and solid circles indicate high and low ration groups, respectively. Dashed and solid lines represent the linear models fitted to the data of high and low ration groups, respectively.

The fecundity of mature females was 6296.5 ± 3452.0 (n = 16, mean ± SD) in the HR and 2251.3 ± 860.0 (n = 13) in the LR groups (Fig 5). All initial explanatory variables were selected in the linear models for log (fecundity) or log (relative fecundity). Exponents of SL to the fecundity (relative fecundity) were estimated to be 4.53 (1.43) and 0.26 (−2.11) in HR and LR groups, respectively (Table 4).

**Table 4:** Results of the linear model for log (fecundity) or log (relative fecundity) of western sand lance (*Ammodytes japonicus*) in Experiment II.

Analysis of variance				Summary			
Error source	SS	df	*P*	Variable	Estimate	SE	*p*
Log (Fecundity)							
Error	2.07	25		Intercept	-11.76	3.00	<0.001
Log (SL)	3.07	1	<0.001	Log (SL)	4.53	0.66	<0.001
Group	1.45	1	<0.001	Group (LR)	18.29	6.11	0.006
Log (SL) : Group	0.79	1	0.005	Log (SL): Group (LR)	-4.28	1.39	0.005
Log (relative fecundity)							
Error	1.32	25		Intercept	1.66	2.40	0.49
Log (SL)	0.093	1	0.19	Log (SL)	1.43	0.53	0.012
Group	1.18	1	<0.001	Group (LR)	15.12	4.88	0.005
Log (SL) : Group	0.54	1	0.004	Log (SL): Group (LR)	-3.54	1.11	0.004

Analysis of variance was operated by Type II F test.

SS: sum of squares.

Initial explanatory variables were log (standard length [SL]), ration group (high ration [HR] and low ration [LR]), and the interaction thereof, and all variables were selected.

The effect of LR group was assessed on the basis of HR group.

## Discussion

To the best of our knowledge, this is the first study to demonstrate that western sand lance adjusts its energy allocation for skeletal and somatic growth in response to food availability before aestivation. YOY sand lance first allocated energy to both growth avenues but thereafter shifted to somatic growth with negligible skeletal growth under low food availability (Fig 1). One possible factor that drove the shift in energy allocation was water temperature. High water temperature increases metabolic costs and, therefore, limits energy allocation to somatic growth. On the other hand, fish under the same conditions but with continuous access to an abundance of food exhibited continuous skeletal growth and entered aestivation earlier than fish with limited access to food. The earlier onset of aestivation may be a defense action to reduce predation risks by decreasing the duration spent swimming and exposed in the water column [39]. Additionally, larger western sand lance enter aestivation earlier in the field [27], which suggests that fish of a smaller size and worse condition may delay their aestivation in attempt to increase their size or weight prior to aestivation. Delayed onset of aestivation under low food availability is expected to result in a lower survival rate, and hence, a reduced reproductive potential of the species.

This study also clearly showed that food availability during even just a few months before aestivation is critical for the winter reproduction in YOY western sand lance. The importance of food availability prior to aestivation for successful reproduction, namely (1) the increase in fecundity, (2) the increase in the number of maturing fish, and (3) the earlier onset of maturation and spawning as revealed in this study, offers new insights into the underlying mechanisms that govern the population dynamics of western sand lance.

A noteworthy finding of this study was that the relative fecundity positively increased with body size in the HR group but not in the LR group (Fig 5). This result indicates that the number of eggs produced by first-time spawning western sand lance largely varies in response to food availability, even under the same spawning stock biomass. Our findings differed from those of Atlantic cod, in which the potential fecundity of first-time spawning fish was not affected by food availability [40]. The fecundity of fish at 80 mm was predicted to be 3321 and 2115 in the HR and LR groups, respectively. Similarly, the fecundity at 90 mm was 5665 and 2180 in the HR and LR groups, respectively. The large difference in fecundity between the ration groups even at the same body size indicates that food availability before aestivation strongly affects the reproductive output. The down regulation caused by the nutritional condition of parental fish at several months before the spawning season has been reported in some capital breeders, such as Atlantic herring [11] and Atlantic cod [41]. Thus, the links between food availability, parental fish condition, and egg production may be common in capital breeders.

Low food availability before aestivation results in an increase in the number of immature fish. Approximately 13–23% of individuals did not mature in the LR group, which indicates that energy accumulation was not sufficient for reproductive investment in this group. The GLM suggested that body size strongly affected the decision of maturation. However, size is not the single factor that determines the maturity of western sand lance, because sufficient somatic condition at aestivation is also necessary [38]. Maturity determination in relation to the body size and condition has been suggested for some capital breeders, such as Atlantic salmon *Salmo salar* [42] and *A*. *marinus* [43]. Notably, body size and condition govern the maturity of the genus *Ammodytes* [33,34,38]. Furthermore, the reduction rate of body weight from before to after aestivation was greater in the LR group. This result may be related to the smaller body size of the LR group because energy consumption owing to the metabolism during aestivation would be larger in smaller individuals.

Low food availability before aestivation also delayed the onset of maturation and the timing of spawning, whereby the maturation rate increased at a later-time (Fig 2). From December to January, the oocyte diameter was smaller in females from the LR group than in those from the HR group (Fig 4). Although the mechanisms of the delay still remain to be elucidated, one possible explanation is that larger-sized individuals have accumulated a sufficient amount of energy to be able to allocate more energy to gonadal development, as inferred from *A*. *marinus* [44]. The timing of spawning affects the timing of larval hatching, which can lead to the match or mismatch with zooplankton blooms: a key factor for recruitment in *A*. *marinus* [45–47]. Thus, food availability before aestivation not only affects the timing of spawning but may also affect the survival of for western sand lance larvae.

The present study revealed that the food availability to YOY fish before aestivation affected several reproductive traits, such as the timing of maturation and the number of offspring. These findings are expected to contribute to the understanding of the underlying mechanisms that govern the variation in the inter-annual recruitment of western sand lance. In the Seto Inland Sea, the condition factor of YOY western sand lance at the beginning of aestivation has decreased in recent years, suggesting the recent decline in the food availability during spring-summer (Nishikawa T. et al., pers.com.). If so, the reproductive potential of YOY, occupying large portion of spawning stock biomass in this water, appears to have been decreased, as inferred from our study. Other factors, such as the variation in temperature, should also be taken into consideration [40]. Rising temperatures can have negative effects on the reproduction of the genus *Ammodytes* [29,31,48]. Furthermore, the body size or condition of parental fish may affect the quality of eggs and larvae [49], and such maternal effects have been reported in some capital breeders, such as chum salmon *Oncorhynchus keta* [50], Atlantic cod [51], and Atlantic salmon [7]. Although differences in egg size and feeding rate of hatched larvae was not detected between the HR and LR groups of western sand lance [38], the maternal effects should be further investigated in future studies.

## Supporting information

S1 Dataset Data of western sand lance in Experiment I. (XLSX)

S2 Dataset Data of western sand lance in Experiment II. (XLSX)
